# Age estimation based on Willems method versus country specific model in Saudi Arabia children and adolescents

**DOI:** 10.1186/s12903-021-01707-9

**Published:** 2021-07-13

**Authors:** Ali Alqerban, Muath Alrashed, Ziyad Alaskar, Khalid Alqahtani

**Affiliations:** 1 Department of Preventive Dental Sciences, College of Dentistry, Prince Sattam bin Abdulaziz University, Riyadh, Saudi Arabia; 2 Department of Preventive Dental Science, Dar Al Uloom University, Alkharj, Saudi Arabia; 3 Department of Oral Health Sciences, KU Leuven & Dentistry, University Hospitals Leuven, Leuven, Belgium; 4 Department of Oral and Maxillofacial Surgery and Diagnostic Sciences, College of Dentistry, Prince Sattam bin Abdulaziz University, AlKharj, Saudi Arabia

**Keywords:** Forensic science, Forensic odontology, Dental age estimation, Tooth development, Willems model, Saudi Arabia

## Abstract

**Background:**

The aims of this study were to create a method for estimation of dental age in Saudi children and adolescents based on the Willems model developed using the Belgian Caucasian (BC) reference data and to compare the ability of the two models to predict age in Saudi children.

**Methods:**

Development of the seven lower left permanent mandibular teeth was staged in 1146 panoramic radiographs from healthy Saudi children (605 male, 541 female) without missing permanent teeth and without all permanent teeth fully developed (except third molars). The data were used to validate the Willems BC model and to construct a Saudi Arabian-specific (Willems SA) model. The mean error, mean absolute error, and root mean square error obtained from both validations were compared to quantify the variance in errors in the sample.

**Results:**

The overall mean error for the Willems SA method was 0.023 years (standard deviation, ± 0.55), indicating no systematic underestimation or overestimation of age. For girls, the error using the Willems SA method was significantly lower but still negligible at 0.06 years. A small but statistically significant difference in total mean absolute error (11 days) was found between the Willems BC and Willems SA models when the data were compared independent of sex. The overall mean absolute error for girls was slightly lower for the Willems BC method than for the Willems SA method (1.33 years vs. 1.37 years).

**Conclusions:**

The difference in ability to predict dental age between the Willems BC and Willems SA methods is very small, indicating that the data from the BC population can be used as a reference in the Saudi population.

## Background

Estimation of dental age is a useful method for determining chronological age [1]. The scientific literature recommends calculation of dental age as the best method for age estimation in children, for whom there is a high correlation between chronological age and tooth growth [2]. Furthermore, dental age estimation has the least error in children [3] and has been found to be more useful than chronological age when planning orthodontic treatment [4].

Radiologically registered dental growth in children can be classified using the staging technique devised in 1973 by Demirjian et al. [5]. This method remains the one most commonly used for age estimation. Using a sample of French Canadian children, Demirjian et al. created a set of growth curves and tables, the validity of which has been confirmed in different populations [6, 7]. They also developed an age estimation method based on the developmental processes that occur in the permanent lower left teeth (excluding the third molar). The methodology developed by Demirjian et al. [5] for estimation of age has since been updated using a large sample of the Belgian Caucasian (BC) population by Willems et al. [8]. Some researchers have suggested that the Willems BC method performs better than other methods for estimation of dental age [2, 9–16], one of the main reasons given being that it is based on a large sample of BC children with a more or less equal age and sex distribution (i.e., 2116 panoramic radiographs for children aged 3–18 years).

In Saudi Arabia, there is an increasing need for age estimation in children because of human trafficking, migration, asylum procedures, child pornography, adoption of children without a birth certificate, and legal decisions. When all seven teeth are available, the Willems approach shows the least difference when dental and chronological age are compared and could be used for age estimation [17]. Furthermore, the Willems method has been validated using multiple country-specific reference data [8]. However, the ability of the Willems method to predict age in Saudi children has not been well studied and it is unclear whether or not there is a need for a Saudi Arabian-specific reference method. Therefore, the present study aimed to validate the Willems BC method in a sample of Saudi children, create a Saudi Arabian-specific (Willems SA) method of age estimation, and to compare the ability of the two methods to predict age in these children.

## Methods

Ethical approval to perform this study was granted by the institutional review board of Dar Al Uloom University in Riyadh, Saudi Arabia (approval number: RCE 0007-2017) and performed in accordance with the Declaration of Helsinki. A retrospective search of patient records at the Dar Al Uloom University Hospital and the School of Dentistry, Prince Sattam bin Abdulaziz University, Al-Kharj, Saudi Arabia for October 2016 to August 2017 yielded 1334 panoramic radiographs for 712 Saudi boys and 622 Saudi girls aged 4–18 years (Table 1). A total of 1146 subjects (605 boys and 541 girls) were < 16 years with no missing permanent teeth and did not have all permanent teeth of the left mandible fully developed. Only those were included in the analysis. Signed informed consent was obtained from each subject’s parents or legal guardians. Children were deemed to be of Saudi ethnicity if the names of both parents were of traditional Saudi origin. Each child’s birth date was registered from their birth certificate or another form of identification provided by their parents or guardians. At both centres, digital panoramic radiographs were acquired by an Orthophos XG5 machine (Sirona Dental Systems, Bensheim, Germany), which includes a digital charge-coupled device line sensor with exposure parameters of 14.1 s, 64 kV, and 8 mA. All images were stored in JPEG file format with a size of 2.5 MB and 2.440 × 1280 pixels.

**Table 1:** Age distribution of sampled Saudi Arabian children

Age	*n*	Mean	SD	Min	Q1	Median	Q3	Max
	1334	10.31	4.00	2.08	7.03	9.52	13.49	17.99
4-4.99	78	4.53	0.31	4.01	4.26	4.54	4.86	5.00
5-5.99	119	5.54	0.32	5.00	5.27	5.60	5.83	6.00
6-6.99	124	6.50	0.32	6.00	6.21	6.50	6.79	7.00
7-7.99	132	7.50	0.30	7.00	7.25	7.51	7.73	8.00
8-8.99	142	8.49	0.32	8.00	8.22	8.49	8.77	9.00
9-9.99	121	9.46	0.30	9.00	9.21	9.46	9.70	10.00
10-10.99	107	10.47	0.30	10.00	10.20	10.43	10.70	11.00
11-11.99	90	11.49	0.32	11.00	11.19	11.56	11.75	12.00
12-12.99	61	12.44	0.28	12.01	12.22	12.46	12.66	12.97
13-13.99	50	13.46	0.28	13.01	13.21	13.50	13.67	13.97
14-14.99	42	14.49	0.31	14.00	14.27	14.49	14.75	14.95
15-15.99	80	15.57	0.30	15.00	15.30	15.63	15.86	16.00
16-16.99	96	16.54	0.31	16.01	16.29	16.57	16.80	17.00
17-17.99	92	17.49	0.28	17.00	17.27	17.48	17.73	17.99

Age: age groups of 1 year; n: total number of subjects per age category; Descriptive statistics per age category of 1 year: mean: mean age; sd: standard deviation; median: median age; min: minimum age; max: maximum age; Q1: ages at percentile 25; Q3: ages at percentile 75

The study inclusion criteria were excellent image quality and no evidence of a developmental dental abnormality in the medical history or on panoramic radiographs [18]. Radiographs in which two bilateral corresponding permanent mandibular teeth (other than the third molars) were absent were excluded [18]. To avoid observer bias, all panoramic radiographs were automatically sorted into numerical order before analysis and all other data were anonymised. The Photoshop® CS2 program (Adobe Systems Inc., San Jose CA, USA) was used for stage allocation and allowed for magnification and enhancement of images when necessary.

**Fig. 1:**
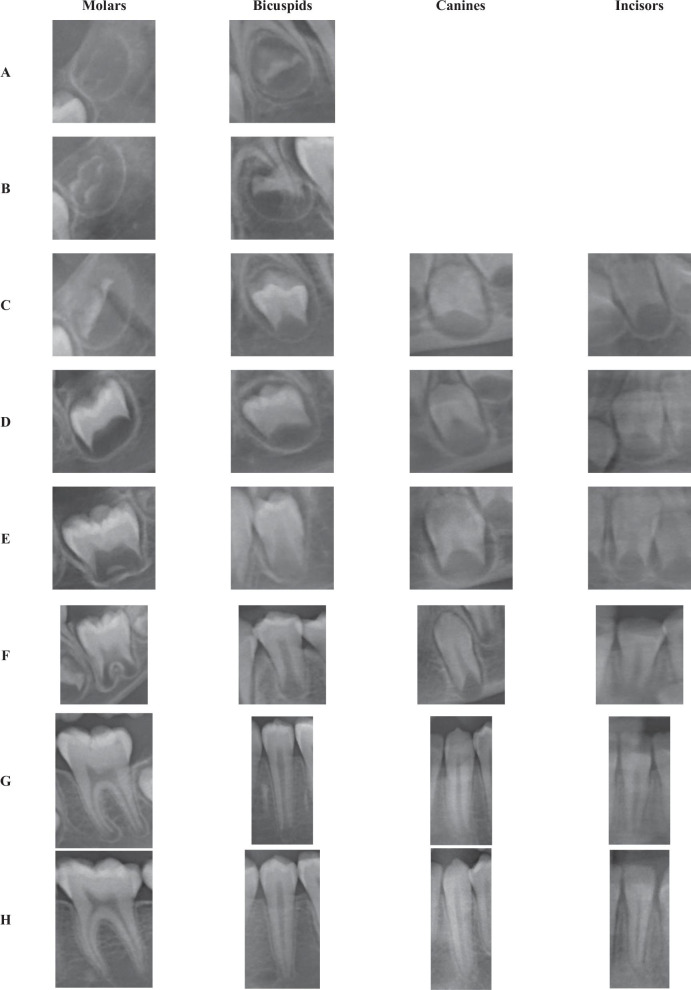
Determining the developmental stage of the seven left permanent mandibular teeth using Demirjian et al. (1973) method on Saudi Arabian sample

All panoramic radiographs were evaluated using the methods described by Demirjian et al. in 1973 and by Willems et al. in 2001 [5, 8]. Only the seven lower left mandibular teeth (except for the third molar) were scored. First, each tooth was graded as ‘A’ to ‘H’ according to calcification stage (Fig. 1). Each score was then translated into a dental age based on sex as defined by Demirjian et al. and Willems et al. [5, 8]. Dental maturity scores were calculated for all seven left mandibular permanent teeth in both sexes using the weighted analysis of variance method used in the Willems BC model [8].

All panoramic radiographs were scored by three examiners. Two weeks later, the same examiners reviewed 100 randomly selected images. Differences between actual and predicted age were determined. The data were randomly categorised but stratified by sex and age into a test dataset and a training dataset. Specific regression coefficients were obtained for Saudi Arabian samples from a fit of the model on the full Saudi Arabian dataset using the Willems BC method [8]. The samples in the training dataset was fitted for a Saudi Arabian-specific model using the methodology of Willems et al. (2001). The test dataset was used to compare and validate the constructed Saudi Arabian-specific prediction model and the model devised by Willems et al. in 2001. The age prediction error was described as the difference between chronological age and estimated age (i.e., chronological age−estimated age) for comparison of the age prediction results. For calibration purposes, the error was expressed as the mean error (ME, representing both overestimations and underestimations) to quantify the direction of the error, mean absolute error (MAE) to quantify the magnitude of the error, and the root mean square error (RMSE) to quantify the variance in errors in the sample (assigning large errors more weight).

The interquartile range (lowest quartile, 25th percentile; highest quartile, 75th percentile) was calculated to concentrate on the data in the middle of the study. The Wilcoxon signed-rank test was used to compare the mean difference between chronological age and estimated age (ME, i.e., bias), the MAE, and the RMSE. Weighted kappa values were used to evaluate the interobserver and intraobserver reliability of the scores. The intraobserver reliability based on the first observer was 0.983 and the interobserver reliability was 0.919, indicating excellent agreement. All statistical analyses were performed using SAS software (version 9.4; SAS Institute Inc., Cary, NC, USA). A *P* value < 0.05 was considered as being statistically significant.

## Results

**Fig. 2:**
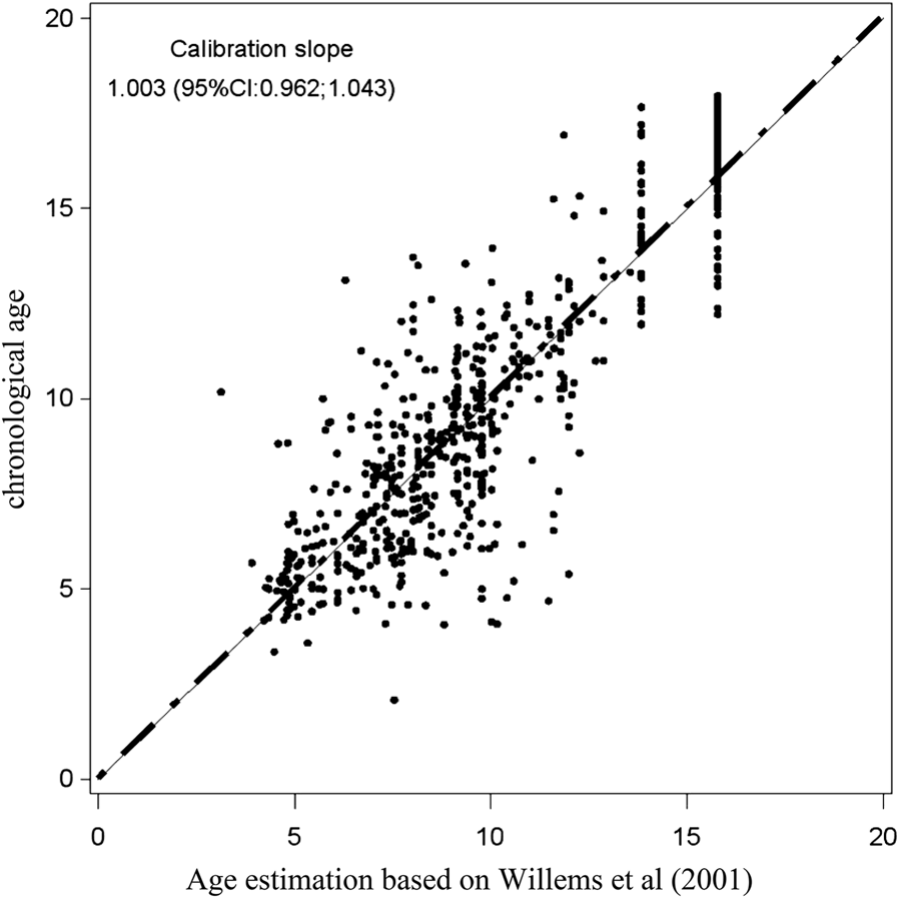
Calibration slope in the plot of chronological age (Willems BC method) against the predicted age (Willems SA method) for Saudi girls. BC, Belgian Caucasian; SA, Saudi Arabian

**Fig. 3:**
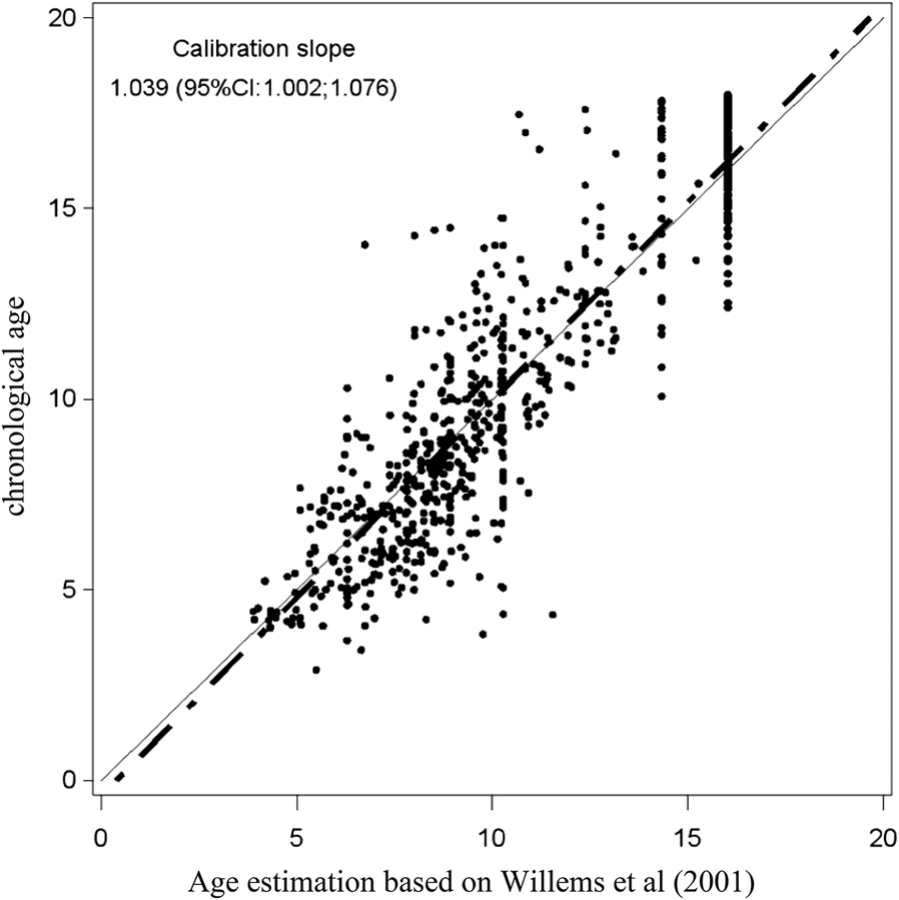
Calibration slope in the plot of chronological age (Willems BC method) against predicted age (Willems SA method) for Saudi boys. BC, Belgian Caucasian; SA, Saudi Arabian

Table 2 shows the developmental stages based on Demirjian et al. (1973) categorised by age. The mean estimated age was not significantly different from the mean chronological age when both sexes were combined. Figures 2 and 3 show the calibration slope in the plot of chronological age (Willems BC method) against predicted age (Willems SA method) separately for girls and boys. There was no significant difference in the calibration slope between the two approaches. A negative value for the mean difference in chronological age against predicted age indicates overestimation and a positive value indicates underestimation of mean age.

**Table 2:** Overall maturity scores for females and males for each of developmental stages as reported by Demirjian et al. (1973)

Scores	Sex	t31	t32	t33	t34	t35	t36	t37
A	F	0.00	0.00	0.00	0.00	0.80	0.00	0.80
	M	0.00	0.00	0.00	0.14	0.70	0.00	1.40
B	F	0.00	0.00	0.16	0.16	1.44	0.16	0.80
	M	0.00	0.00	0.00	0.28	1.82	0.42	1.54
C	F	0.00	0.16	0.48	4.00	3.36	0.00	2.08
	M	0.14	0.14	0.42	3.77	4.47	0.00	2.51
D	F	2.88	2.72	6.88	8.16	8.96	1.76	22.08
	M	2.23	2.51	6.56	9.22	8.94	1.96	23.18
E	F	9.76	12.32	25.12	31.20	33.12	11.68	28.48
	M	11.87	14.25	29.05	33.52	32.96	12.15	28.49
F	F	12.32	17.12	28.00	21.28	21.44	6.72	14.24
	M	10.75	15.36	27.79	18.58	18.30	6.42	11.87
G	F	31.04	25.12	13.12	9.60	6.72	37.12	12.48
	M	30.17	25.14	9.22	7.40	7.82	34.36	10.34
H	F	44.00	42.56	26.24	25.60	24.16	42.56	19.04
	M	44.83	42.60	26.96	27.09	25.00	44.69	20.67

N: number; M: male; F: Female; t: tooth

On average, the estimated age was 0.02 years (standard deviation [SD] 1.78) higher than the chronological age, but was not statistically significant. Although there was no bias in boys, there was a small but negligible difference between predicted age and chronological age in girls, the predicted age being on average 0.06 years (SD 1.80) higher and − 0.01 years (SD 1.76) in boys (Table 3). The MAE was 0.02 years and was similar for boys and girls (1.34 years vs. 1.33 years), resulting in a mean RMSE for the total sample of 1.77 years (95 % confidence interval 1.71–1.84; Table 3).

**Table 3:** Mean error and absolute mean error and root mean squared error and 95 % confidence intervals validating the Willems et al. (BC) method, overall and sex specific

*Sex*	*N*		Mean	SD	*P*
M + F	1146	Chronological Age	10.31	4.00	0.6653
		Estimated age	10.28	3.51	
		Error	0.02	1.78	
		RMSE (95 %Cl)	1.7780 (1.711;1.845)	–	
M	605	Chronological Age	10.50	4.04	0.5055
		Estimated age	10.50	3.50	
		Error	− 0.01	1.76	
		Absolute Error	1.34	1.13	
		RMSE (95 %Cl)	1.7582 (1.667;1.849)	–	
F	541	Chronological Age	10.09	3.96	0.1560
		Estimated age	10.03	3.51	
		Error	0.06	1.80	
		Absolute Error	1.33	1.22	
		RMSE (95 %Cl)	1.8003 (1.700;1.900)	–	

N: Number; M: total number of male subjects; F: total number of female subjects; Descriptive statistics for chronological age based on the method by Willems et al. [11] and for the (absolute) error between chronological age and estimated age: mean age and standard deviation (SD); RMSE: root mean squared error; 95 %CI: 95 % confidence intervals for RMSE; P: p-value from Wilcoxon signed rank test comparing age and age estimation based on Willems et al. (2001) [3]

Table 4 shows the regression coefficients for both sexes. The test dataset validated the efficiency of both the Willems BC method (5) and the newly developed Willem SA method. Table 5 demonstrates the mean difference (error), the absolute mean difference, the proportion of subjects with a predicted age within one year of chronological age, and a comparison of the mean RMSE for both strategies. Overall, the MAE and RMSE were slightly lower (albeit not significantly) using the Willems BC method. The relationship between age and error was comparable in magnitude using both approaches. The age of older subjects tended to be underestimated whereas that of younger subjects tended to be overestimated. The overall MAE for the Willems SA method was slightly higher than that for the Willems BC method (1.36 years vs. 1.33 years). There was also a slight variation in MAE for both approaches in both sexes.

**Table 4:** Saudi Arabian specific regression coefficients for males and females separately obtained from a fit of the model on the full Saudi Arabian dataset according to the Willems BC method (2001)

	Tooth	B	C	D	E	F	G	H
M	31	−	6.141	4.656	5.847	5.383	5.874	5.556
	32	−	0.000	0.000	− 0.702	− 0.226	0.083	0.562
	33	−	0.000	0.213	0.633	1.244	1.518	1.763
	34	0.011	1.189	1.162	0.638	0.974	2.475	2.707
	35	-0.293	− 0.390	− 0.456	0.214	0.399	0.612	2.147
	36	0.000	−	− 1.486	− 1.252	− 1.130	− 0.413	− 0.198
	37	0.160	0.522	0.992	1.514	1.872	2.555	3.654
F	31	−	−	11.204	11.403	12.426	12.948	11.855
	32	−	0.000	− 0.084	0.000	− 0.601	− 0.349	0.165
	33	0.000	-4.567	− 5.126	− 5.176	− 4.347	− 3.385	− 3.024
	34	0.000	0.000	0.279	0.308	0.412	0.886	1.976
	35	− 1.355	-0.376	− 0.869	− 0.513	− 0.325	− 0.103	1.999
	36	0.000	−	0.000	− 0.300	0.032	0.266	0.661
	37	− 0.095	− 0.221	0.134	0.589	0.503	0.899	2.578

M: male; F: Female

**Table 5:** Differences in mean error, mean absolute error, and root mean squared error; between Willems et al. (BC) method and Willems et al. (SA) method

Sex	*N*	*§*	Mean (CI%)	SD	*P*
M + F	1146	Error Willems et al. BC method	0.02	1.77	
		Error Willems et al. SA method	0.00	1.80	
		Difference	0.02	0.55	0.1183
		Absolute error Willems et al. BC method	1.33	1.16	
		Absolute error Willems et al. SA method	1.37	1.18	
		Difference	− 0.03	0.52	0.1775
		RMSE Willems et al. BC method	1.778 (1.71;1.85)		
		RMSE Willems et al. SA method	1.803 (1.74;1.87)		
F	605	Error Willems et al. BC method.	0.05	1.78	
		Error Willems et al. SA method	0.00	1.81	
		Difference	0.04	0.60	0.0107
		Absolute error Willems et al. BC method	1.32	1.20	
		Absolute error Willems et al. SA method	1.37	1.18	
		Difference	− 0.05	0.55	0.0927
		RMSE Willems et al. BC method	1.800 (1.70;1.90)		
		RMSE Willems et al. SA method	1.809 (1.71;1.91)		
M	541	Error Willems et al. BC method.	− 0.01	1.76	
		Error Willems et al. SA method	0.00	1.80	
		Difference	− 0.01	0.51	0.2690
		Absolute error Willems et al. BC method	1.35	1.13	
		Absolute error Willems et al. SA method	1.37	1.17	
		Difference	− 0.02	0.49	0.5646
		RMSE Willems et al. BC method	1.758 (1.67;1.85)		
		RMSE Willems et al. SA method	1.799 (1.71;1.89)		

Willems SA method newly constructed dental age estimation method using the Willems et al. [3] methodology on a Saudi Arabian reference database and validated. RMSE: root mean squared error; CI%: 95 % confidence intervals for the RMSE are given between brackets; N: number; M: male; F: Female; SD: standard deviation, P: P-value from Wilcoxon signed rank test comparing Absolute error Willems et al. and Absolute error Willems SA method

## Discussion

This research was performed to evaluate the population-specific weighted score that needs to be applied when the Willems BC method is used for estimation of dental age in Saudi children. The differences in ME, MAE and RMSE between the prediction from the Willems BC method and Willems SA method reflect the usefulness of the Belgian population as a reference.

Overestimation of dental age in relation to chronological age has been found in many studies. In a study performed in Malaysian children by Cherian et al. (2020), age was overestimated in children aged 6–15 years by an average of 0.04 ± 1.08 years in boys and by 0.03 ± 1.18 years in girls [19]. Furthermore, in a study of children in Bosnia and Herzegovina by Galic et al. (2011), age was overestimated by an average of 0.42 years in boys and by 0.24 years in girls [11]. Nevertheless, underestimation has been reported by other authors. Cameriere et al. (2008) applied the Willems method to children in Italy, Spanish and Croatia and observed that the age of girls was underestimated by age 0.07 and males overestimated by age 0.25 [20]. In addition, a study performed on central southern Chinese Han population aged 8–16 years by Yang et al. (2019), there was underestimation in females by 0.54 years and 0.44 years for males [21]. A possible explanation for the differences between overestimation or underestimation of dental age in relation to chronological age might be attributed to the different ethnic groups. The difference between boys and girls is most likely biological as often observed for the entire growth period between boys and girls. In this study, girls indicated advanced dental development and reached dental age maturation earlier than boys. Saudi Arabia’s legal system applies judicial punishment differently starting at the age of seven than it does at lower ages. A mother’s custody of her son and daughter ends when they reach the ages of 11 and 13, respectively. At the age of 15, permission to work is granted. People under the age of 18 may be committed to a rehabilitation center during their judgment.

In a study of Saudi boys and girls aged 8.5–17 years, Al Emran et al. (2008) noted that dental age was slightly older than chronological age by an average of 0.3 years in boys and 0.4 years in girls [22]; moreover, a study by Baghdadi (2013) found a mean difference of 0.77 ± 0.85 years in boys and 0.85 ± 0.79 years in girls [23]. A similar study by Qudeimat and Behbehani (2009) in Kuwaiti children aged 3–14 years found that dental age was overestimated by 0.71 ± 1.18 years in boys and by 0.67 ± 1.30 years in girls [24]. Our present findings in girls are consistent with the results of the above-mentioned three studies, but not for boys. However, a study in the Western Saudi population by Alshihri et al. in 2016 showed that Saudi girls were 0.059 ± 1.25 years and Saudi boys were 0.66 ± 1.14 years ahead of French Canadian children [25]. These findings indicate that genetic differences between Arab and European populations do not have a significant effect on dental growth or estimation of age and that the Willems model can be used to estimate age in Saudi children.

The Willems BC approach has not been validated or developed as a prediction model in the Saudi population. However, it was anticipated to perform well in Saudi children [26] based on the findings of studies in Japan [3], the United Arab Emirates [27], Brazil [28], and Malaysia [29], in which the Willems BC method was found to be suitable in those population samples. While a small overestimation of chronological age has been reported for the Willems BC method, both this method and the recently developed Malaysian-specific model have estimated age of similar magnitude and error variance. Our results confirm that the Willems BC method is accurate in Saudi children. Moreover, our findings are consistent with those of several previous studies [9, 14, 30]. Willems et al. found that the overall MAE was slightly higher with the Willems BC method than with a South African-specific method in black children (0.68 years vs. 0.62 years) [18]. However, Cadenas de Llano-Pérula et al. and Metsäniitty et al. found that the overall MAE was same the Willems BC method [31, 32]. Our result shows that the overall MAE to be slightly higher when using the Willems BC method than when using the Willems SA method (1.37 years vs. 1.33 years) and that the MAE was the same in boys and girls (1.37 years). We also found a small but statistically significant difference in the total MAE (11 days) and RMSE (11 days) for both sexes between the Willems BC and Willems SA methods (Table 5). However, our average age estimates for both sexes in a sample of the Saudi population are not significantly different from those estimated by the Willems BC method [8] (Table 3).

This research has some limitations that should be borne in mind when interpreting its results. The first is that the performance of the Willems BC method [8] was based on a broad reference database (n = 2116) whereas that of the Willems SA method was based on a smaller country-specific database (n = 1146). Furthermore, neither the Demirjian method nor the Willems method can be used in children with hypodontia in the mandible. A relative limitation of the study was that the subjects included here had undergone dental panoramic radiography for valid clinical reasons related to dental health and deviations from normal occlusal development, which explains the difference in sample numbers across the age groups.

In future research both permanent teeth and third molars might be integrated with the same model in order to produce an age estimation model that addresses most of the present shortcomings, especially within the ranges 15–17.99 years. The largest error in age estimation at present is this transition point between two age estimation approaches because the number of useful age-related parameters present is less.

## Conclusions

In Saudi children, there is no need to use a Saudi Arabian-specific model instead of the Belgian reference database when estimating age. The difference in mean absolute error for age prediction was close enough to zero (0.03) to be considered clinically irrelevant. There was no systematic underestimation or overestimation of age. Although age estimation was significantly less accurate in girls, at 22 days it was still negligible in magnitude.

## Data Availability

The datasets used and analysed during the current study are available from the corresponding author on reasonable request.

## Abbreviations

BC: Belgian Caucasian
SA: Saudi Arabian
ME: Mean error
MAE: Mean absolute error
RMSE: Root mean square error
SD: Standard deviation
